# In Vitro Testing of Crude Natural Plant Extracts from Costa Rica for Their Ability to Boost Innate Immune Cells against *Staphylococcus aureus*

**DOI:** 10.3390/biomedicines5030040

**Published:** 2017-07-05

**Authors:** Ragheda Yaseen, Katja Branitzki-Heinemann, Hani Moubasher, William N. Setzer, Hassan Y. Naim, Maren von Köckritz-Blickwede

**Affiliations:** 1 Department of Physiological Chemistry, University of Veterinary Medicine Hannover, Bünteweg 17, 30559 Hannover, Germany; ragheda.yaseen@tiho-hannover.de (R.Y.); Katja.Branitzki-Heinemann@tiho-hannover.de (K.B.-H.); Hassan.Naim@tiho-hannover.de (H.Y.N.); 2 Botany & Microbiology Department, Cairo University, Giza 12613, Egypt; moubasher@sci.cu.edu.eg; 3 Department of Chemistry, University of Alabama in Huntsville, Huntsville, AL 35899, USA; setzerw@uah.edu; 4 Research Center for Emerging Infections and Zoonoses (RIZ), University of Veterinary Medicine Hannover, Bünteweg 17, 30559 Hannover, Germany

**Keywords:** monocytes, neutrophil, neutrophil extracellular trap, *Byrsonima crassifolia*, *Mandevilla veraguasensis*, *Verbesina oerstediana*

## Abstract

The increasing occurrence of antibiotic-resistant *Staphylococcus* (*S*.) *aureus* tremendously limits the antibiotic-based treatment options; therefore, an open discussion of alternative treatment strategies is urgently needed. The use of naturally derived materials might become a more promising concept, not only as directly acting antimicrobials, but also for stimulation of the immune system. Costa Rican plant extracts were screened for their ability to enhance the antimicrobial activity of human blood-derived cells against *S. aureus* infections. We identified three plant extracts which significantly reduced the growth of *S. aureus* in the presence of human blood without directly acting as antibacterials: *Byrsonima crassifolia* acetone bark extract, *Mandevilla veraguasensis* acetone vine extract and *Verbesina oerstediana* acetone bark extract (VEOEBA). The effect of VEOEBA was studied in more detail, and revealed that VEOEBA increases the antimicrobial activity of neutrophils by enhancing the formation of neutrophil extracellular traps.

## 1. Introduction

*Staphylococcus (S.) aureus* is a Gram-positive bacterium which is well-known for causing a variety of infections including skin infections, pneumonia, endocarditis, osteomyelitis, respiratory infections, and food poisoning [1,2]. In addition to being the causative agent of several life-threatening diseases, *S. aureus* is an important zoonotic pathogen, and because of the emergence of antibiotic resistance, is nowadays a major healthcare issue [3]. Since there is an increasing prevalence of methicillin-resistant *S. aureus* (MRSA), the classical antibiotic-based treatment will become even more limited [4,5].

The modern use of naturally derived materials might be “old-fashioned”, but can be a promising concept against antibiotic-resistant bacteria. Naturally derived products, such as plant extracts, have become the focus of attention not only because they may directly act as an antimicrobial against the pathogen, but they may also be able to boost the host immune system against an infection. Interestingly, we have recently shown that acetone bark extract from *Guarea kunthiana* (GUKUBA), at a concentration of 1 μg/mL, has the ability to boost the antimicrobial activity of human and bovine neutrophils against bacterial infections [6].

Cells of the innate immune system successfully fight against invading pathogens and thereby efficiently protect the host against infectious microorganisms. Circulating leukocytes, such as monocytes and neutrophils, belong to the first line of defense, and are equipped with numerous antimicrobial mechanisms, such as phagocytosis and cytokine production [7]. Discovered in 2004, neutrophil extracellular traps (NETs) have been additionally described as a novel phagocytosis-independent antimicrobial mechanism of neutrophils. Those traps consist of nuclear DNA, and are released by the cells into the extracellular milieu to entrap and kill bacteria [8]. Interestingly, the natural product anacardic acid from cashew nut shells has recently been shown to stimulate NETs production, and thereby the overall bactericidal activity of immune cells [9].

Thus, development of drugs from natural products that boost the antimicrobial activity of host innate immunity may help against antibiotic resistant pathogens. Therefore, 23 plant extracts derived from Costa Rica were screened to investigate their ability to enhance the antimicrobial activity of blood-derived immune cells against staphylococcal infections (see Table 1).

## 2. Experimental Section

### 2.1. Preparation of Plant Extracts

The plant material was collected in May 2003 from the Monteverde Cloud Forest Reserve, Costa Rica. The trees were identified by William A. Haber, and voucher specimens have been deposited in the Missouri Botanical Garden Herbarium. The material was chopped and air-dried; dried plant material was extracted with refluxing solvent using a Soxhlet extractor for 4 h. The solvent was evaporated to give crude extract. For further experiments, the crude extract was dissolved in dimethyl sulfoxide (DMSO) to 10 mg/mL per stock, and tested at a final concentration of 10 μg/mL, or a lower concentration, as indicated in the figure legends.

### 2.2. Bacteria

The experiments herein utilize *Staphylococcus aureus* strain Newman, which was originally isolated in 1952 from a patient suffering from tubercular osteomyelitis [10]. *S. aureus* Newman was grown in brain heart infusion (BHI) medium at 37 °C with shaking. Overnight cultures were diluted 1:100 in BHI and grown to logarithmic growth phase (OD_600_ = 0.5). Bacterial suspensions were diluted in respective cell culture media to the desired concentration at a multiplicity of infection (MOI) of 2:1 colony forming units (cfu) per cell.

### 2.3. Isolation and Preparation of Monocytes and Neutrophils

Human monocytes were isolated from fresh blood of healthy voluntary donors by density gradient centrifugation using the PolymorphPrep™ system (Axis-Shield), as previously described [11], and in agreement with the local ethical board. The upper layer with monocytes was transferred into a fresh, sterile 50 mL tube, and washed with PBS before the cells were resuspended in RPMI + 2% fetal calf serum. Neutrophils were harvested and used as previously described [6].

### 2.4. Whole Blood, Monocyte and Neutrophil Killing Assay

The experiment was performed as earlier described [6]. Briefly, bacteria were grown until logarithmic phase. Fresh human blood from healthy voluntary donors, isolated monocytes or neutrophils were incubated with 1:120 diluted *S. aureus* (blood) or an MOI of 2 (neutrophils or monocytes) for defined time periods in the presence or absence of natural products, at a concentration of 10 μg/mL. Blood was incubated under rotation at 37 °C and 5% CO_2_. Neutrophils and monocytes were incubated without rotation at 37 °C and 5% CO_2_. In control experiments, bacteria were grown in BHI without blood or cells to determine the direct antibacterial effect of the plant extracts. Surviving bacteria were plated in serial dilutions on Todd Hewitt broth (THB) agar and enumerated after overnight incubation at 37 °C. The percentage of surviving bacteria was calculated in comparison with a bacterial growth control that was grown under the same conditions, except in the absence of cells. The survival factor was calculated relative to time point 0 (min after infection), set as 1.

### 2.5. Neutrophil Extracellular Traps Induction Assay

The capacity of blood-derived neutrophils to form NETs was assessed after stimulation with phorbol myristate acetate (PMA) as a positive control, VEOEBA, or vehicle control. Cells were seeded on 12-mm coverslips coated with poly-l-lysine, stimulated with selected reagents, and centrifuged for 5 min at 472× *g*. The plates were then incubated at 37 °C and 5% CO_2_ in a humidified incubator for 2, 4 or 6 h. Cells were fixed by addition of paraformaldehyde (PFA) in phosphate buffered saline (PBS) to a final concentration of 4% PFA. For all conditions, preparations were performed in duplicate.

### 2.6. NET Visualization and Quantification

The experiment was performed as described earlier [6]. Briefly, fixed cells were washed with PBS, permeabilized and blocked with 2% bovine serum albumin (BSA) in 0.2% Triton X-100/PBS. NETs were stained with a first mouse monoclonal anti-H2A-H2B-DNA complex antibody (PL2–6, 0.5 μg/mL), and a secondary antibody Alexa Fluor 488 rabbit anti-mouse IgG (1:500, Invitrogen, Carlsbad, CA, USA). After washing, slides were mounted in Prolong Gold with 4′,6′-diamidino-2-phenylindole (DAPI) (Invitrogen). Images were recorded using a Leica TCS SP5 confocal microscope with a HCX PL APO 40× (0.75–1.25 numerical aperture) oil immersion objective. Settings were adjusted with control preparations using an isotype control antibody (mouse IgG2b). For each preparation, three randomly selected images were acquired and used for quantification of NET-producing cells. Data were expressed as percentages of NET-forming cells.

### 2.7. Cytotoxicity Test

Freshly isolated neutrophils were treated with VEOEBA crude acetone bark extract (10 μg/ mL) for 90 min and 4 h incubation at 37 °C, 5% CO_2_. Samples were stained with 0.4 mg/mL trypan blue as indicator for dead cells; based on their positive staining, the percentage of dead cells was calculated compared to total cell count, using light microscopy.

### 2.8. Statistical Analysis

Data were analyzed by using a paired, one-tailed *t*-test (GraphPad Software). Experiments were performed as at least at 3 independent occasions. Values less than 0.05 were considered significant (* *p* < 0.05, ** *p* < 0.005, *** *p* < 0.001).

## 3. Results and Discussion

### 3.1. Effect of Plant Extracts on Antimicrobial Activity of Blood

In the actual study, we tested 23 plant extracts (Table 1) for their ability to boost the antimicrobial activity of fresh human blood against *S. aureus* (Figure 1 and Figures S1–S20). We identified three plant extracts that significantly reduce the growth of *S. aureus* in the presence of human blood without directly acting as antibacterials (Figure 1). Bacteria were incubated with and without plant products for various time points in the presence (Figure 1) or absence (Figure 2) of whole human blood, and then plated to check for surviving bacterial counts. BYCRBA, MAVEVA and VEOEBA (see Table 1) were able to boost the antimicrobial activity of human blood against *S. aureus* at a final concentration of 10 μg/mL, at the time points 90 and 120 min (Figure 1). All other plant extracts listed in Table 1 showed no significant effect (Figures S3–S22).

In control experiments, *S. aureus* was co-incubated with the different plant extracts at 37 °C in the absence of blood (Figure 2). Staphylococcal growth was monitored by plating surviving bacteria on agar plates to enumerate bacterial numbers. Interestingly, the extracts themselves exhibited no significant direct antimicrobial impact on the bacteria (Figure 2). Only a slight effect on bacterial growth was visible with MAVEVA (Figure 2b).

### 3.2. Effect of Plant Extracts on Antimicrobial Activity of Monocytes and Neutrophils

**Figure 1:**
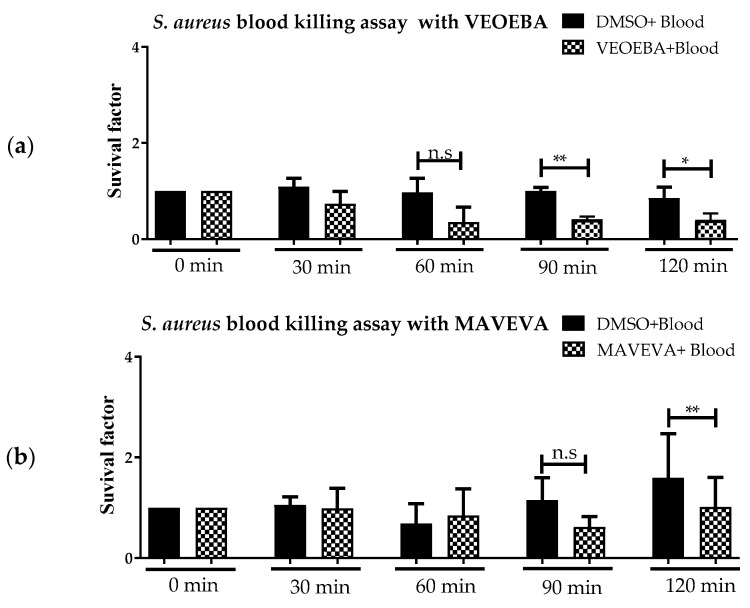

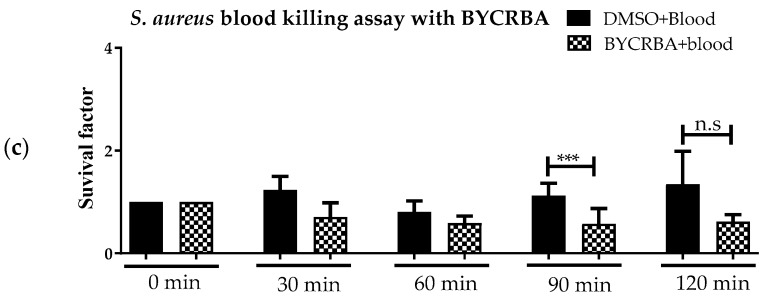
BYCRBA (**a**), MAVEVA (**b**), and VEOEBA (**c**) boost whole blood antimicrobial activity against *S. aureus*. Significant reduction of the bacterial count at time points 90 min and 120 min compared to non-treated cells was recorded after treatment of fresh human blood with the different plant extracts at a concentration of 10 μg/mL. Depicted are the bacterial survival factors at defined time points in comparison to *t* = 0 from a minimum of *n* = 3 independent experiments. ** *p* ˂ 0.005, *** *p* < 0.001 one-tailed, paired Student’s *t*-test. Dimethyl sulfoxide (DMSO).

**Figure 2:**
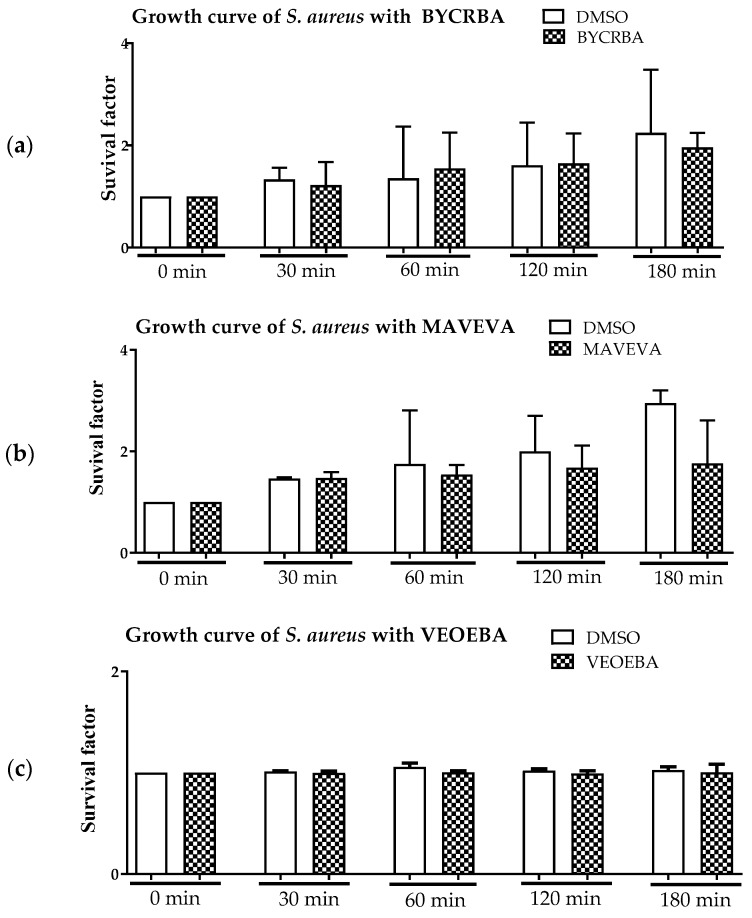
BYCRBA (**a**), MAVEVA (**b**), and VEOEBA (**c**) show no direct antimicrobial effect against *S. aureus*. Depicted are the bacterial survival factors at defined time points in comparison to *t* = 0 from *n* = 3 independent experiments.

**Figure 3:**
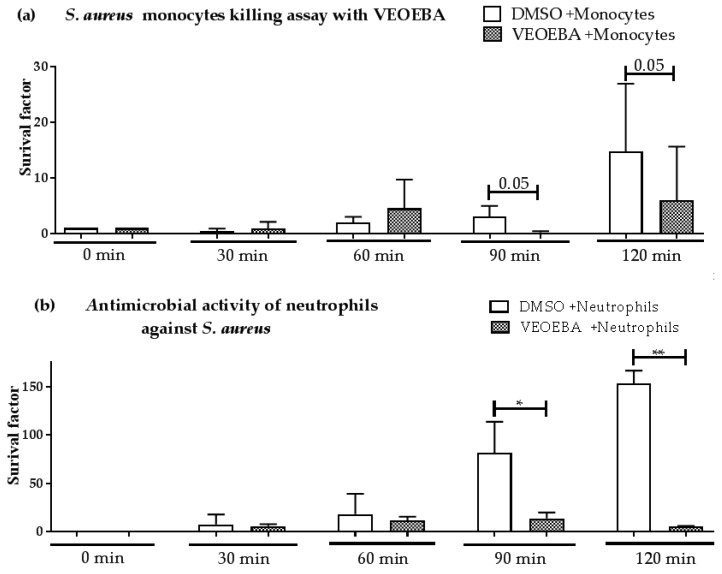
VEOEBA enhances the antimicrobial effect of monocytes (**a**) and neutrophils (**b**) against *S. aureus*. Depicted are bacterial survival factors at defined time points compared to *t* = 0 from a minimum of *n* = 3 independent experiments. * *p* < 0.05 one-tailed, paired Student’s *t*-test.

The most promising candidate with highest the most pronounced effect on bacterial growth in Figure 1, VEOEBA, was studied in more detail: human monocytes and neutrophils were pre-stimulated with VEOEBA or vehicle control and were analyzed for their capability to fight *S. aureus* over time. Indeed, VEOEBA enhances the antimicrobial activity of both immune cell types, as monitored by a reduction of surviving bacteria (Figure 3).

### 3.3. Effect of Plant Extracts on Formation of Neutrophil Extracellular Traps (NETs)

**Figure 4:**
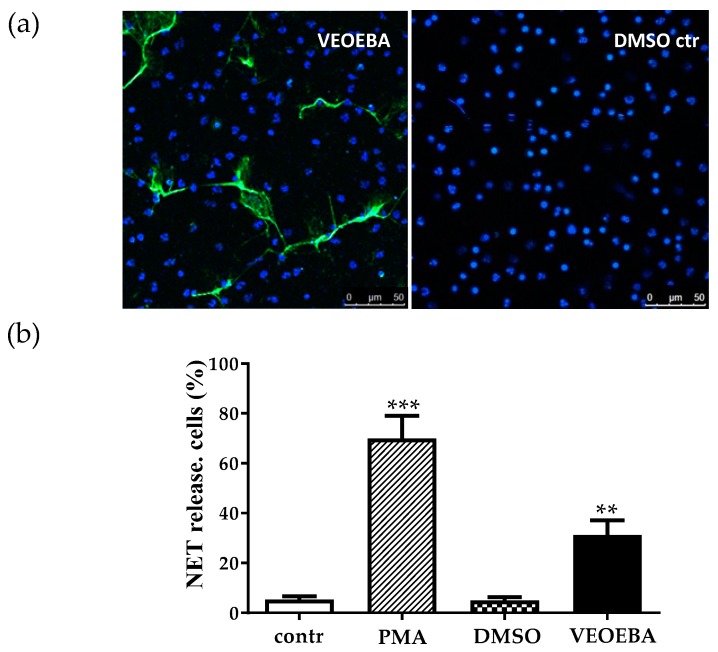
VEOEBA significantly induces the NET formation of human neutrophils after 4 h of incubation, compared to DMSO control. Depicted in (**b**) are the percentages of NET-releasing cells compared to total amount of neutrophils from a minimum of *n* = 4 independent experiments. Representative images are shown in (**a**). NETs were visualized using double-staining with 4′,6′-diamidino-2-phenylindole (DAPI) to stain DNA (**blue**), and monoclonal mouse anti-H2A-H2B-DNA complex antibody followed by an Alexa 488-rabbit anti-mouse antibody (**green**). ** *p* < 0.005, *** *p* < 0.001 one-tailed, paired Student’s *t*-test.

A very important defense mechanism of neutrophils against *S. aureus* is the formation of NETs. NETs consist of extracellularly released NET-like fibers which mainly consist of DNA-histone complexes, which are able to entrap and kill various bacteria [8]. Several authors have shown that NETs act efficiently as antimicrobials against *S. aureus* [8,11,12]. Since there are also some natural products found among the wide class of NET-inducing stimuli [6,9], it was interesting to investigate the NET-stimulating potential of VEOEBA. As shown in Figure 4, VEOEBA significantly induces the number of NET-releasing neutrophils compared to the vehicle control DMSO after 4 h of incubation, though to a lesser extent than the positive control (PMA-stimulated cells).

**Figure 5:**
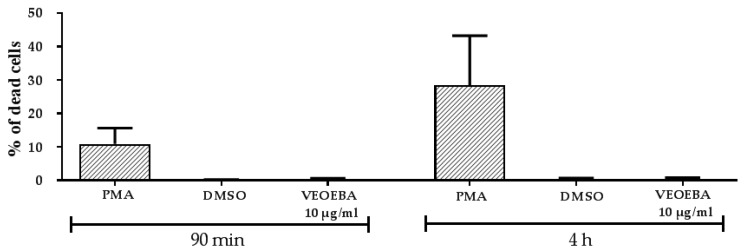
VEOEBA does not induce the death of neutrophils after 90 min or 4 h of incubation, tested by trypan blue staining and subsequent quantification of the stained cells compared to total amount of neutrophils per field of view, from *n* = 3 independent experiments. * *p* < 0.05 one-tailed, unpaired Student’s *t*-test.

Most publications describe NET release as a form of pathogen-induced active cell death, which gives neutrophils the possibility to fight against microbes beyond their life span [13]. However, it was recently demonstrated that neutrophils released NETs during an acute infection with *S. aureus*, and those neutrophils were viable and still able to phagocytose and crawl [12]. Thus, a suicidal and a vital NET-formation can be differentiated [12,13,14]. Interestingly, incubation of neutrophils with VEOEBA had no cytotoxic effect on the neutrophils as shown by trypan blue staining, whilst PMA-stimulation tremendously increased the number of dead cells, similar to previous publications [8,11,13] (Figure 5). These data indicate that VEOEBA induces the viable type of NET-formation.

## 4. Conclusions

Concluding, the presented results show that out of 23 plant extracts, three (BYCRBA, MAVEVA and VEOEBA) were able to significantly boost the antimicrobial activity of human blood against *S. aureus* at a concentration of 10 μg/mL. The concentration was chosen based on preliminary data received with GUKUBA [6], and based on the testing of different concentrations for two selected plant extracts BYCRBA and VEOBA (Figure S21). All plant extracts are from plants known as traditional medicinal plants: BYCRBA (*Byrsonima crassifolia*) was shown to act as an antidepressant with additional anti-inflammatory activity and gastro-protective effects [15,16]. The seed of BYCRBA affects diabetes as it decreases blood glucose [17]. Interestingly, the ethyl acetate of roots from BYCRBA showed antimicrobial effect against *Klebsiella pneumoniae*, *Pseudomonas aeruginosa*, *Salmonella typhi*, *Shigella flexnari*, *S. aureus*, *S. epidermis*, *Streptococcus pneumoniae* and *Micrococcus luteus* [18]. Importantly, BYCRBA extracts have been shown to be rich in bioactive polyphenolics, such as vitexin [19]. However, the antimicrobial or neutrophil-modulating compound in BYCRBA extracts is still not characterized. To our knowledge, no direct antimicrobial activity of the plant extracts VEOEBA (*Verbesina oerstediana*) or MAVEVA (*Mandevilla veraguasensis*) have been previously described. Those plant extracts are traditionally used against diabetes (VEOEBA) [20] or serve as medical remedy against snake bites (MAVEVA) [21].

Focusing on VEOEBA as an example, we could identify that this bark extract is able to increase the antimicrobial activity of monocytes and neutrophils, which are key effector cells of the innate immune response machinery against *S. aureus*. Moreover, VEOEBA stimulates the release of NET-structures without significantly affecting the viability of the cells. This is an interesting phenotype since boosting of formation of NETs has been proven to be protective against severe *S. aureus*-mediated pneumonia in vivo [11]. However, details on the underlying mechanisms of the VEOBEBA-mediated NET-formation are still unclear and need further investigation. Furthermore, intense research on the identification of active compounds is still lacking and needs to be done in the future.

Overall, the results support the hypothesis that stimulating the immune system might improve the host defense efficiency against invading pathogens, such as *S. aureus*, and may support the conventional antibiotic-based treatments, or offer a new strategy against antibiotic-resistant bacteria. Natural plant products might be promising candidates to search for new therapeutic or prophylactic strategies.

## Figures and Tables

**Table 1:** Plant extracts tested for the ability to boost the antimicrobial activity of human blood against *Staphylococcus (S.) aureus.*

Name	Plant	Solvent	Origin
BRINBC	*Bravaisia integerrima*	chloroform	bark extract
BRINBM	*Bravaisia integerrima*	methanol	bark extract
BYCRBA	*Byrsonima crassifolia*	acetone	bark extract
CESOPA	*Centropogon solanifolius*	acetone	plant extract
DRALBA	*Drypetes alba*	acetone	bark extract
EUELBM	*Euphorbia elata*	methanol	bark extract
MATAYBA	*Matayba oppositifolia*	acetone	bark extract
MAVEVA	*Mandevilla veraguasensis*	acetone	vine extract
OCSIBA	*Ocotea sinuata*	acetone	bark extract
PAMABC	*Paullinia* cf. *macrocarpa*	chloroform	bark extract
PAMABM	*Paullinia* cf. *macrocarpa*	methanol	bark extract
PIAELA	*Piper aequale*	acetone	leaf extract
TALOBC	*Tabernaemontana longipes*	chloroform	bark extract
TALOBM	*Tabernaemontana longipes*	methanol	bark extract
TALOLC	*Tabarnaemontana longipes*	chloroform	leaf extract
TALOLM	*Tabernaemontana longipes*	methanol	leaf extract
TAMEBC	*Tapirira mexicana*	chloroform	bark extract
TRMABA	*Trichilia martiana*	acetone	bark extract
VEOEBA	*Verbesina oerstediana*	acetone	bark extract
VEOELA	*Verbesina oerstediana*	acetone	leaf extract
VETUBA	*Verbesina turbacensis*	acetone	bark extract
VIVELC	*Viburnum venustum*	chloroform	leaf extract
ZABRBA	*Zanthoxylum* sp. “brillante”	acetone	bark extract

## Supplementary Materials

Supplementary materials can be found at http://www.mdpi.com/2227-9059/5/3/40/s1.
