# Supplementary material for: In Vitro Testing of Crude Natural Plant Extracts from Costa Rica for Their Ability to Boost Innate Immune Cells against Staphylococcus aureus
Source: Biomedicines. 2017 Jul 5;5(3):40. doi: 10.3390/biomedicines5030040 (PMC5618298; doi:10.3390/biomedicines5030040)
Supplement: Supplementary file 1 [file biomedicines-05-00040-s001.pdf]

biomedicines

MDPI

In Vitro Testing of Crude Natural Plant Extracts from Costa Rica for Their Ability to Boost Innate Immune Cells against *Staphylococcus aureus*

Ragheda Yaseen, Katja Branitzki-Heinemann, Hani Moubasher, William N. Setzer, Hassan Y. Naim and Maren von Köckritz-Blickwede

Supplemental Figures 1-20: *S. aureus* blood killing assay in the presence or absence of natural plant extracts not shown in Figure 1.

Supplemental Figure 21: *S. aureus* blood killing assay in the presence or absence of different concentration of BYCRBA (a) and VEOBA (b) at 90 min of co-incubation of fresh human blood with the bacteria and the plant extracts.

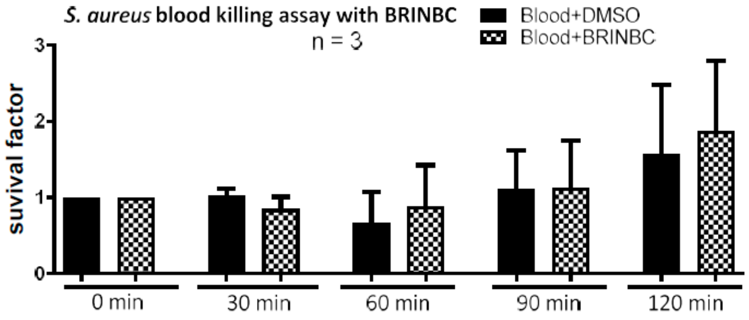

Figure S1.

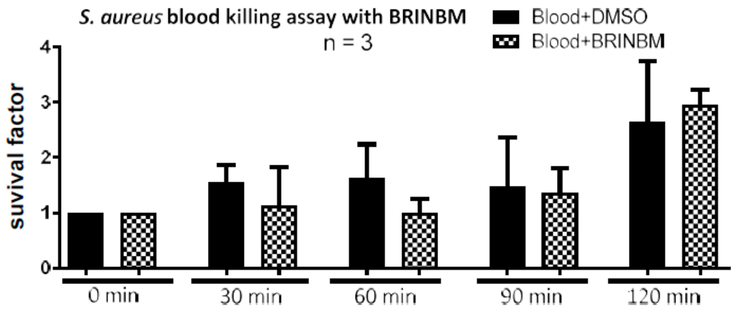

Figure S2.

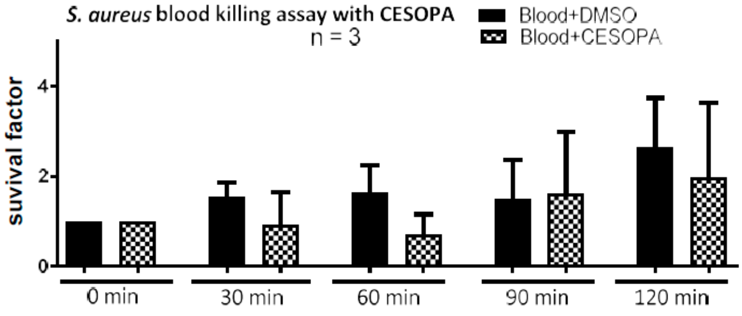

Figure S3.

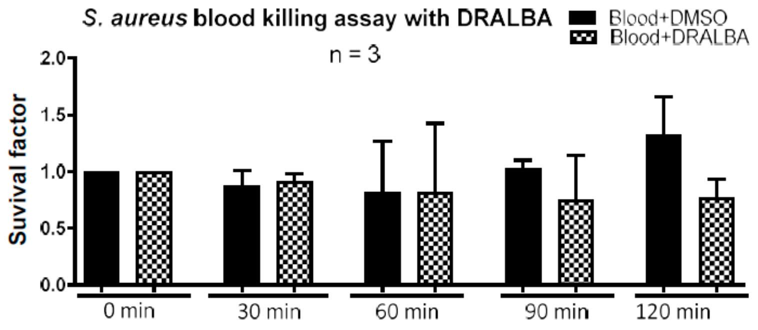

Figure S4.

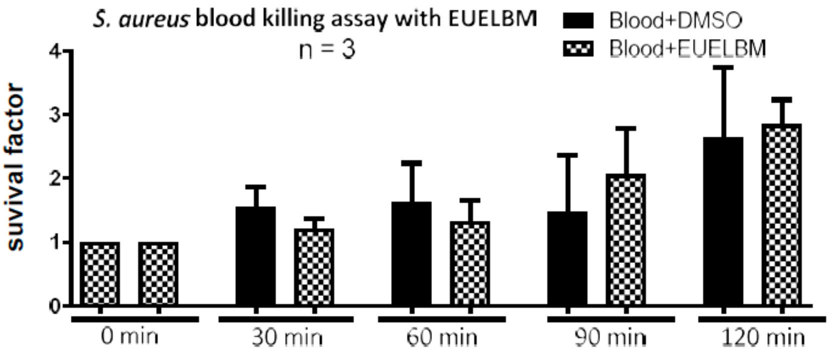

Figure S5.

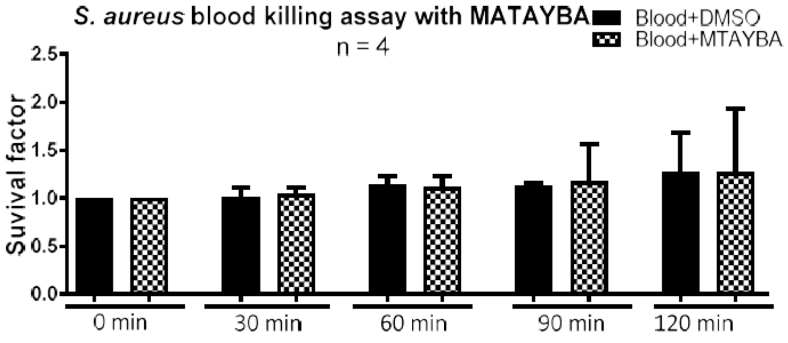

Figure S6.

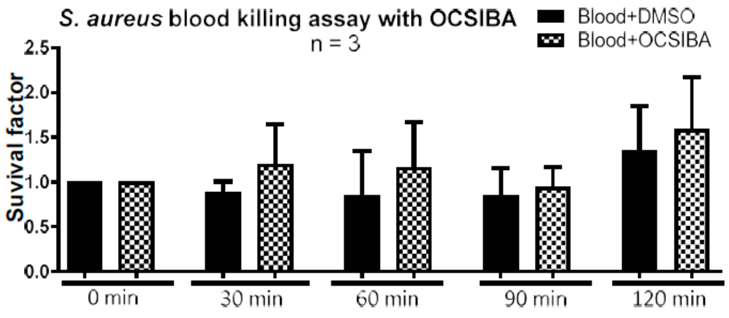

Figure S7.

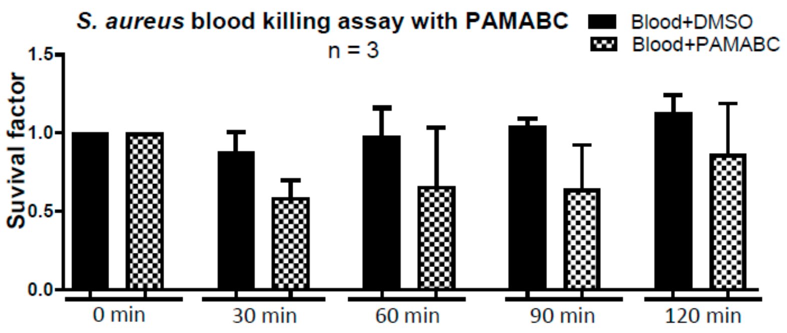

Figure S8.

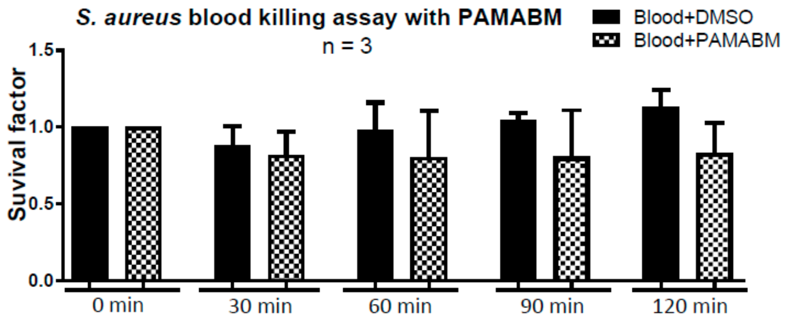

Figure S9.

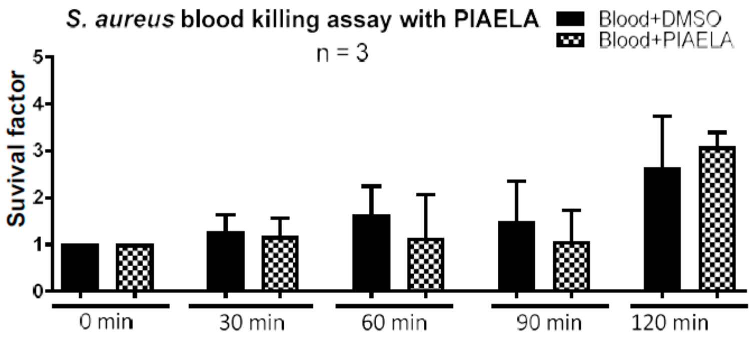

Figure S10.

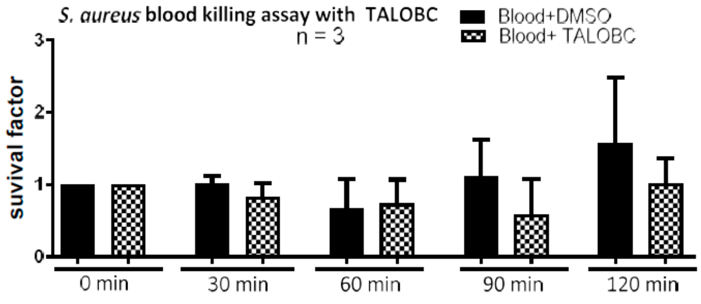

Figure S11.

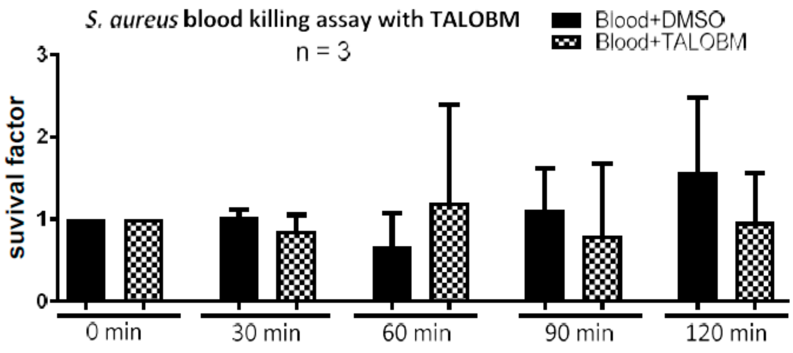

Figure S12.

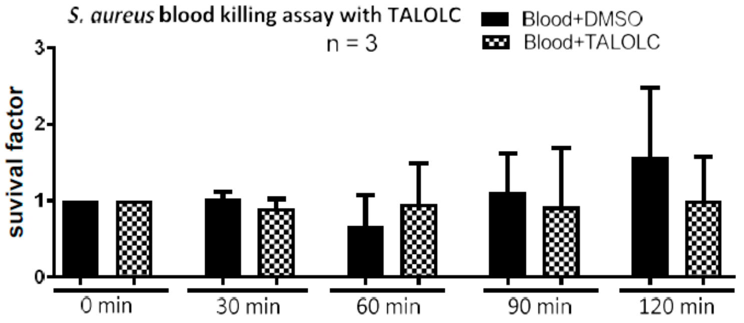

Figure S13.

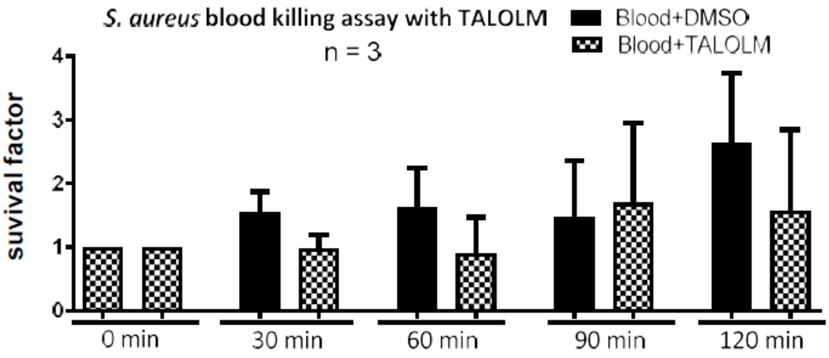

Figure S14.

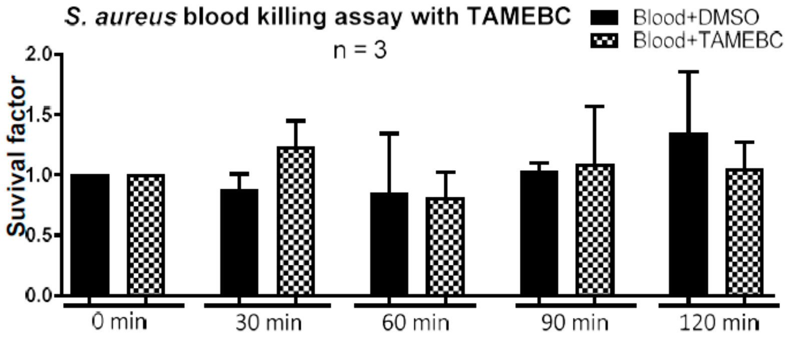

Figure S15.

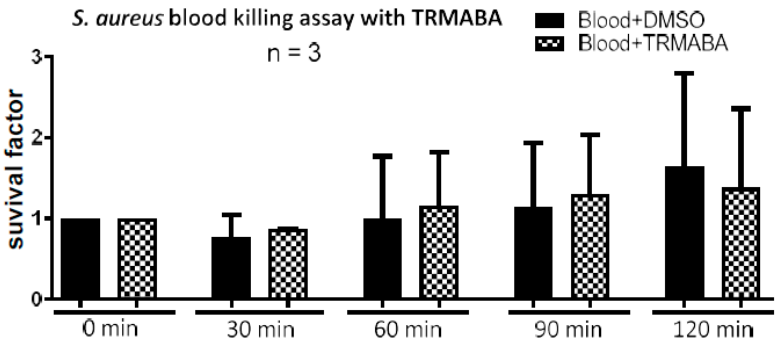

Figure S16.

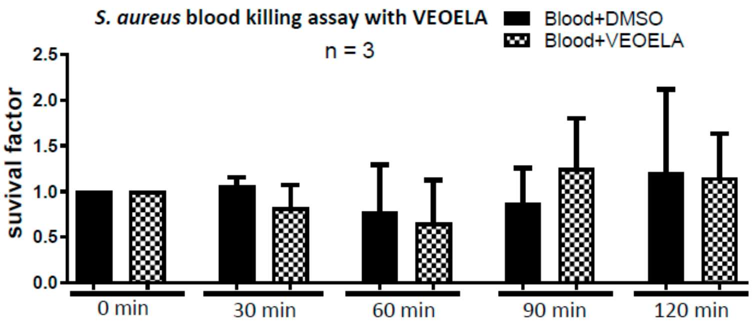

Figure S17.

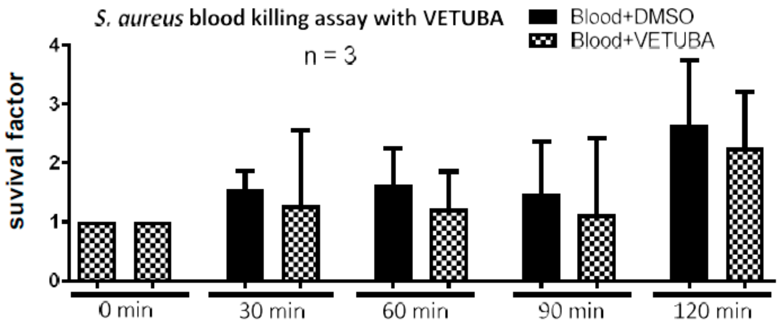

Figure S18.

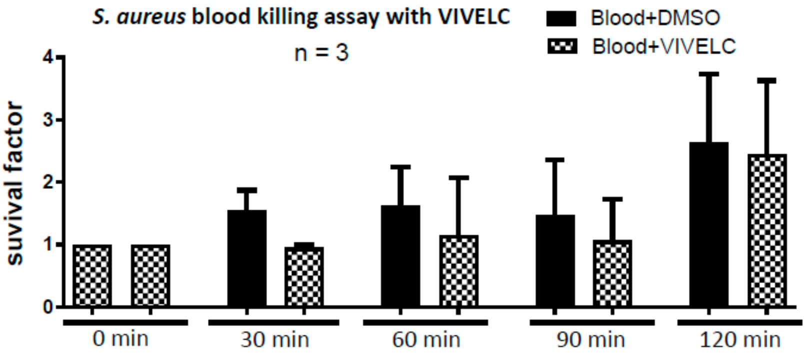

Figure S19.

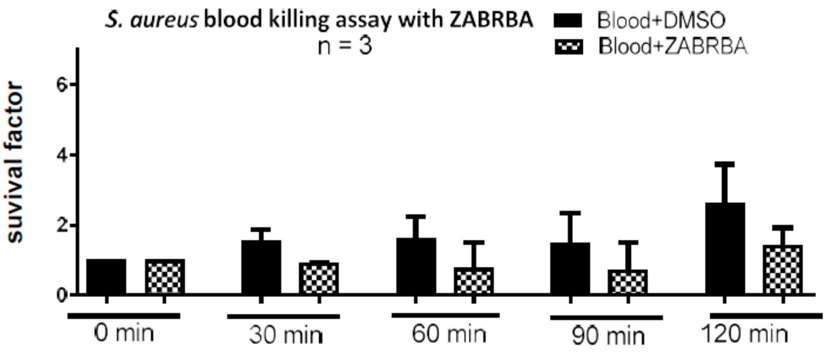

Figure S20.

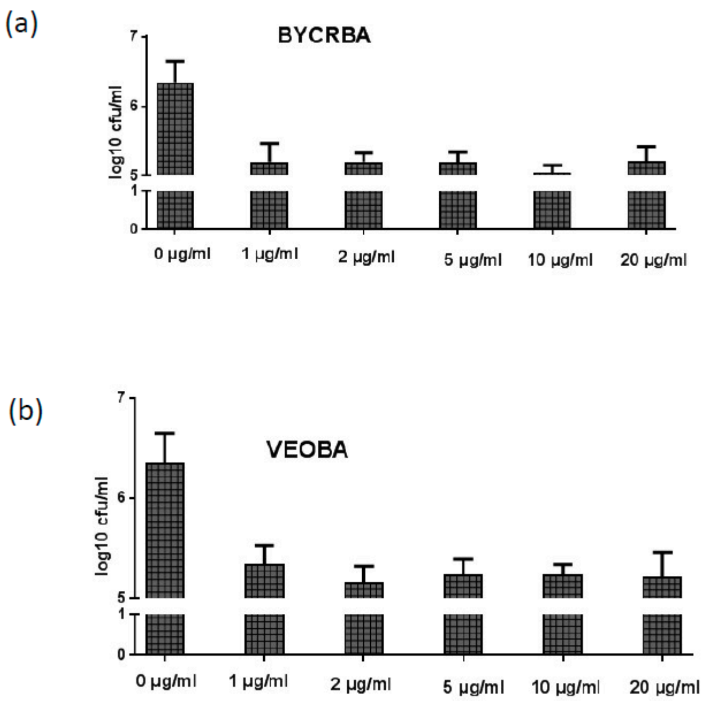

Figure S21.
